# Installing xylose assimilation and cellodextrin phosphorolysis pathways in obese *Yarrowia lipolytica* facilitates cost-effective lipid production from lignocellulosic hydrolysates

**DOI:** 10.1186/s13068-023-02434-9

**Published:** 2023-11-29

**Authors:** Yiran Zhang, Moying Li, Rui Zhu, Yu Xin, Zitao Guo, Zhenghua Gu, Zhongpeng Guo, Liang Zhang

**Affiliations:** 1 National Engineering Research Center for Cereal Fermentation and Food Biomanufacturing, Jiangnan University, Wuxi, Jiangsu 214122 People’s Republic of China; 2 Jiangsu Provincial Engineering Research Center for Bioactive Product Processing, Jiangnan University, 1800 Lihu Avenue, Wuxi, Jiangsu 214122 People’s Republic of China; 3 Yixing Institute of Food and Biotechnology Co., Ltd, Yixing, 214200 People’s Republic of China; 4 School of Food and Biological Engineering, Jiangsu University, Xuefu Road 301, Jingkou District, Zhenjiang, Jiangsu 212013 People’s Republic of China

**Keywords:** Lignocellulosic biomass, Xylose, Cellodextrins, Oleaginous yeast, Lipids

## Abstract

**Background:**

*Yarrowia lipolytica*, one of the most charming chassis cells in synthetic biology, is unable to use xylose and cellodextrins.

**Results:**

Herein, we present work to tackle for the first time the engineering of *Y. lipolytica* to produce lipids from cellodextrins and xylose by employing rational and combinatorial strategies. This includes constructing a cellodextrin-phosphorolytic *Y. lipolytica* by overexpressing *Neurospora crassa* cellodextrin transporter, *Clostridium thermocellum* cellobiose/cellodextrin phosphorylase and *Saccharomyces cerevisiae* phosphoglucomutase. The effect of glucose repression on xylose consumption was relieved by installing a xylose uptake facilitator combined with enhanced PPP pathway and increased cytoplasmic NADPH supply. Further enhancing lipid production and interrupting its consumption conferred the obese phenotype to the engineered yeast. The strain is able to co-ferment glucose, xylose and cellodextrins efficiently, achieving a similar μ_max_ of 0.19 h^−1^, a q_s_ of 0.34 g-s/g-DCW/h and a Y_X/S_ of 0.54 DCW-g/g-s on these substrates, and an accumulation of up to 40% of lipids on the sugar mixture and on wheat straw hydrolysate.

**Conclusions:**

Therefore, engineering *Y. lipolytica* capable of assimilating xylose and cellodextrins is a vital step towards a simultaneous saccharification and fermentation (SSF) process of LC biomass, allowing improved substrate conversion rate and reduced production cost due to low demand of external glucosidase.

**Supplementary Information:**

The online version contains supplementary material available at 10.1186/s13068-023-02434-9.

## Background

The use of renewable resource such as lignocellulosic biomass (or LC biomass) as feedstock for industrial activities will play an essential role for establishing a more sustainable society [1]. While hemicellulose degradation mainly releases xylose, the enzymatic hydrolysis of cellulose only generates hexose. Many of the commercially interest fungal cellulases display low β-glucosidase activity, rendering the hydrolysis of cellodextrins especially cellobiose a rate-limiting step during enzymatic hydrolysis of LC biomass [2]. To achieve efficient LC biomass conversion, different strategies have been employed in recent years. These include metabolic engineering of the production microorganisms [3, 4], engineering of the cellulases [5, 6], strain evolution [7], and the design and construction of artificial microbial consortia [8], etc.

The unconventional yeast *Yarrowia lipolytica* is an attractive workhorse for a variety of applications in detergent, food and pharmaceutical industries [9]. Advantageously, its extraordinary ability to accumulate high cellular content of lipids (more than 30% of its dry cell weight) [10] and its “Generally Recognized as Safe” (GRAS) status, have made this yeast an outstanding host for the production of commercially-useful lipids [11–13]. Nevertheless, despite these advantages, native strain of *Y. lipolytica* is unable to use xylose and cellodextrins as carbon sources. Therefore, engineering *Y. lipolytica* capable of assimilating xylose and cellodextrins is a vital step towards a simultaneous saccharification and fermentation (SSF) process of LC biomass, allowing improved substrate conversion rate and reduced production cost due to low demand of external glucosidase.

In this respect, examples of recent work performed on *Y. lipolytica* are noteworthy. A xylose-fermenting *Y. lipolytica* was constructed by introducing *Scheffersomyces stipitis* xylose reductase (*Ss*XR) and xylitol dehydrogenase (*Ss*XDH), plus overexpressing the endogenous xylulokinase (*Yl*XK). The resulting strain was able to produce citric acid and lipid from xylose [14]. We also made the first attempt to develop an engineered strain of *Y. lipolytica* to co-ferment cellobiose and xylose [15]. However, the xylose-fermenting *Y. lipolytica* demonstrated a preferred glucose utilization over xylose. Also, the reported consumption rates of cellobiose and xylose, and the lipid production yield of the engineered strain were inferior to those obtained in glucose fermentation.

**Fig. 1:**
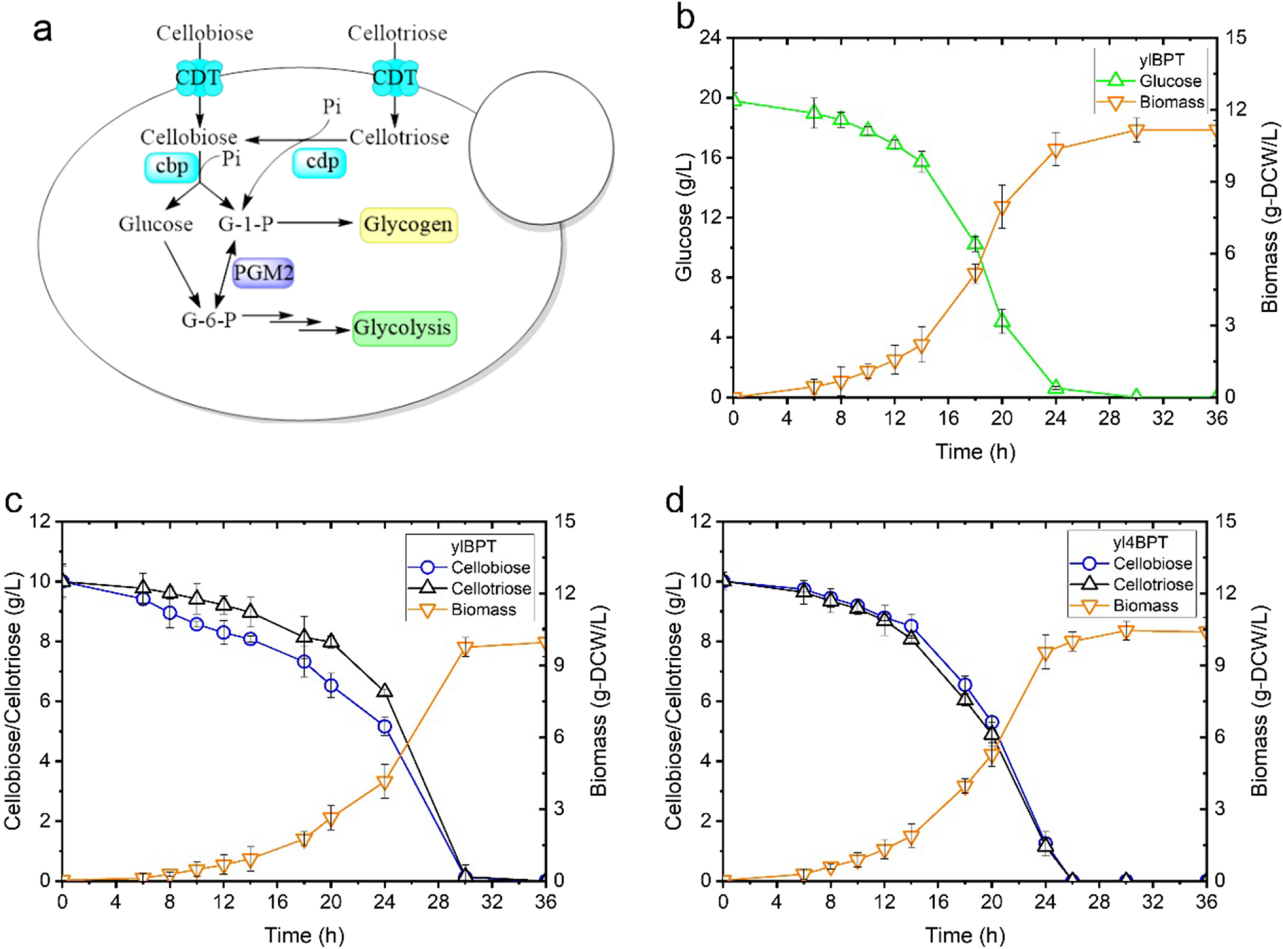
The contrustion of cellodextrin-phosphorololytic *Y*. *lipolytica* strains and their evluation. **a** the schematic diagram of the strategies used in strain engineering. Heterologous genes overexpressed included those encoding: *CDT* cellodextrin transporter, *cbp* cellobiose phosphorylase, *cdp* cellodextrin phosphorylase, *PGM* phosphoglucomutase, Comparison of the growth of (**b**) ylBPT on glucose, (**c**) ylBPT on cellobiose and cellotriose, and (**d**) yl4BPT on cellobiose and cellotriose. Shown are biomass, glucose, cellobiose, and cellotriose concentration versus time. Each data point represents the mean of three independent experiments and the error bar indicates the standard deviation

Herein, we present work to tackle for the first time the engineering of *Y. lipolytica* to produce lipids from cellodextrins and xylose by employing rational and combinatorial strategies. First of all, the construction of a cellodextrins-phosphorolytic *Y. lipolytica* was achieved by expressing the *Neurospora crassa* cellodextrin transporter (*Nc*Cdt1) [16], *Clostridium thermocellum* cellobiose/cellodextrin phosphorylase (*Ct*Cbp/*Ct*Cdp) [17], and the *S. cerevisiae* phosphoglucomutase (*Sc*Pgm2p) [15] (Fig. 1). This strain is capable of cellodextrin-phosphorolysis and glucose-1-phosphate (Glc-1P) production, and is expected to display an energetic advantage over the β-glucosidase-producing strains, since less ATP is consumed in glucose phosphorylation in glycolysis [18]. Then, xylose-fermenting ability was introduced into the above strain by expressing the three key genes in xylose assimilation pathway (*SsXR*, *SsXDH* and *YlXK*), and the gene *CiGXF1* for xylose uptake. Finally, the obese phenotype was conferred to the engineered *Y. lipolytica* by overexpressing the genes *GPD1* and *DGA2*. Gpd1p is involved in the production of precursor for TAG [19], while Dga2p catalyses the synthesis of TAG [20]. In addition, the genes involved in lipid degradation were interrupted. Both xylose assimilation and lipids production were enhanced by increasing the cytoplasmic NADPH supply via the overexpression of the genes *ZWF1* and *GND1*, encoding glucose-6-phosphate dehydrogenase and 6-phosphogluconate dehydrogenase, respectively, in the pentose phosphate pathwapy (PPP). This work paved way for the development of the engineered strains of *Y. lipolytica* to produce valuable chemicals from lignocellulosic hydrolysates at low cost for the advanced generation biorefinery.

## Results

### Expression of cellodextrin transporter and cellodextrin phosphorylase in *Y. lipolytica*

To confer cellodextrin-phosphorolytic capacity to *Y. lipolytica*, the genes *Ctcdp* and *NcCDT1* were introduced into *Y. lipolytica* Po1f (Fig. 1a). The Ura^+^ transformants were selected for their ability to grow on cellobiose, cellotriose and cellotetrose. The results revealed that yeast ylPT co-expressing *NcCDT1* and *Ctcdp* can grow on cellobiose, cellotriose, but not on cellotetrose. Therefore, cellobiose and cellotriose were chosen as carbon sources for the following studies. In addition, the mono-transformant ylP (containing *Ctcdp* only) cannot grow in these conditions, despite the fact that phosphorylase activity was detectable (0.56 ± 0.04 U/mg-total protein on cellobiose and 0.19 ± 0.02 U/mg-total protein on cellotriose). Most likely, *Y. lipolytica* is unable to transport cellodextrins into the cell, or at least the rate of transport (and thus the feed rate to the phosphorylases) was too low to support the growth on these substrates.

The recombinant strains were further characterized in shake flask cultures. The results showed that the growth of ylPT (harboring the cellodextrin phosphorolysis pathway) on cellobiose was extremely poor, and only 4.0 g/L cellobiose was consumed over 36 h at a specific consumption rate of 0.17 g/g-DCW/h (Fig. 1, Table 1). Although it grew better on cellotriose, a lag phase of 12 h was detected. As a result, 7.5 g/L of cellotriose was consumed over 36 h and thereafter, the substrate remained unchanged despite prolonged incubation. The μ_max_ and biomass yield of ylPT on cellotriose was calculated as 0.11 h^−1^ and 0.42 g-DCW/g-cellotriose, respectively, which was 58% and 78% of those obtained on glucose (a μ_max_ of 0.19 h^−1^ and a biomass yield of 0.54 g-DCW/g-glucose) (Fig. 1, Table 1).

**Table 1:** Comparison of the growth of recombinant strains of *Y. lipolytica* on glucose and cellodextrins

Parameter	ylPT			ylBPT		yl4BPT	
Substrate	Glucose	Cellobiose	Cellotriose	Cellobiose	Cellotriose	Cellobiose	Cellotriose
μ_max_ (h^−1^)	0.20 ± 0.01	0.06 ± 0.02	0.11 ± 0.01	0.14 ± 0.02	0.15 ± 0.01	0.17 ± 0.02	0.18 ± 0.01
q (g-s/g-DCW/h)	0.36 ± 0.03	0.17 ± 0.02	0.26 ± 0.03	0.29 ± 0.02	0.30 ± 0.01	0.30 ± 0.01	0.33 ± 0.02
Y_X/S_ (g-DCW/g-s)	0.55 ± 0.02	0.34 ± 0.02	0.42 ± 0.02	0.48 ± 0.01	0.50 ± 0.00	0.52 ± 0.02	0.55 ± 0.02
Fermentation time (h)	24	36	36	36	30	30	26
Residual substrate	0	15.9 ± 1.0	12.6 ± 0.5	0	0	0	0

± the standard deviation. The concentration of cellobiose or cellotriose in culture media was equivalent to that of 20 g/L glucose after hydrolysis

**Fig. 2:**
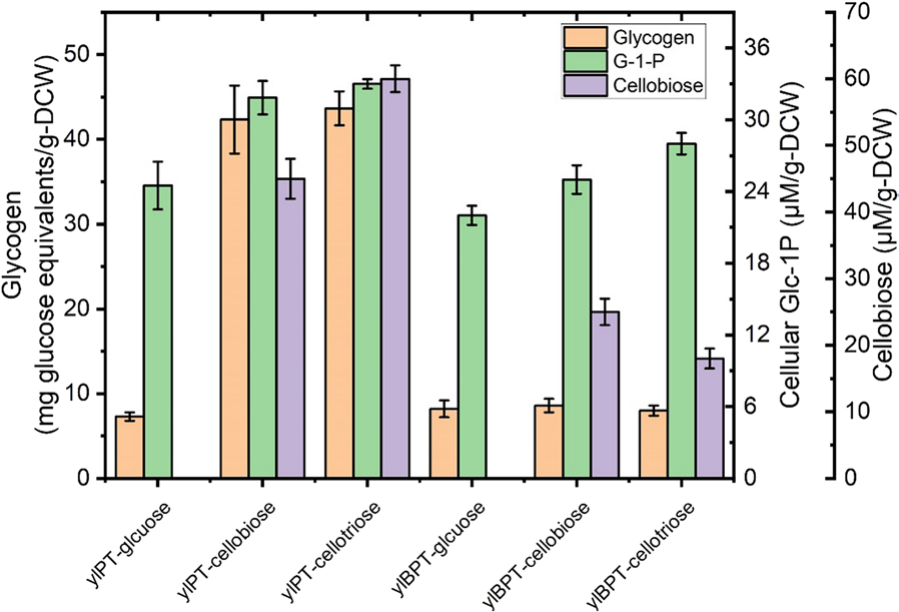
Comparsion of cellular concentration of glycogen, Glc-1P and cellobiose in phosphorolytic *Y. lipolytica* ylPT (*pTEF*-*cdp1*, *pTEF*-*CDT1*) compared with *Y. lipolytica* ylBPT (*pTEF-NcCDT1, pTEF-Ctcdp, pTEF-Ctcdp, pTEF-ScPGM2*) in areobic growth on 20 g/L glucose, or cellobiose or cellobiose. Samples were taken from the cells grown at the exponential phase

A previous work demonstrated that the low conversation of Glc-1P to Glc-6P, which resulted in Glc-1P accumulation and thus a greater flux towards glycogen synthesis, impeded the cellobiose phosphorolysis of an engineered yeast [15]. To investigate the limitations that characterize the phosphorolytic assimilation of cellodextrin in present study, the cellular concentrations of Glc-1P and the reserve carbon source of ylDPT grown on cellotriose were measured. The results showed that ylPT contained 30% more cellular Glc-1P and 5 times more glycogen than when it was grown on glucose (Fig. 2). Strikingly, this analysis also revealed the accumulation of intracellular cellobiose up to 20.0 mg/g-DCW in ylPT. Therefore, poor growth of ylPT on cellobiose is likely due to the low cellobiose phosphorolysis efficiency of Ctcdp. Moreover, cellobiose is also the primary product of cellotriose phosphorolysis whose accumulation may inhibit the following phosphorolysis reaction. Thus, the limiting factors of cellodextrin assimilation are the low conversion rate from Glc-1P to Glc-6P, and the low efficiency in the use of the released cellobiose (Fig. 1).

### Optimization of the cellodextrin-phosphorolytic pathway in *Y. lipolytica*

To tackle the issue of low growth rate of ylPT on cellobiose and -triose due to the Glc-1P accumulation, the gene *PGM2* encoding phosphoglucomutase (PGM) in *S. cerevisiae*, which showed the preference in catalyzing the reaction from Glc-1P to Glc-6P [15], was introduced into ylPT. In addition, the cellobiose phosphorylase of *C. thermocellum* was expressed in ylPT to enhance cellobiose assimilation. The performance of the newly engineered strain ylBPT was evaluated in cellobiose and cellotriose fermentations. As illustrated in Fig. 1, ylBPT demonstrated a 12 h lag phase and a μ_max_ of 0.14 h^−1^ when it was grown on cellobiose. As a result, it consumed all of the cellobiose (20 g/L) over 30 h at a specific consumption rate of 0.29 g/g-DCW/h. Similar results were obtained when the same strain grown on cellotriose. This was accompanied by the decreased cellular content of glycogen and cellobiose resulted from the increased conversion rate of Glc-1P to Glc-6P, which would provide thermodynamic ‘pull’ for cellodextrin phosphorolysis (Fig. 2).

Our last effort was to combine the genes *Ctcbp*, *Ctcdp* and *NcCDT1*, and overexpress them under 4UASTef promoter, aiming to construct a recombinant strain that is able to ferment a mixture of cellobiose and cellotriose efficiently. The resulting strain yl4BPT demonstrated a shortened lag phase of 6 h, a μ_max_ of 0.18 h^−1^ and a biomass yield of 0.54 g-DCW/ g-cellobiose or g-cellotriose, and thus a shorter fermentation time (24 h), all of which were similar to those values of the same strain grown on glucose (Fig. 1, Table 1).

### Engineering *Y. lipolytica* capable of fermenting xylose efficiently in the presence of glucose

**Fig. 3:**
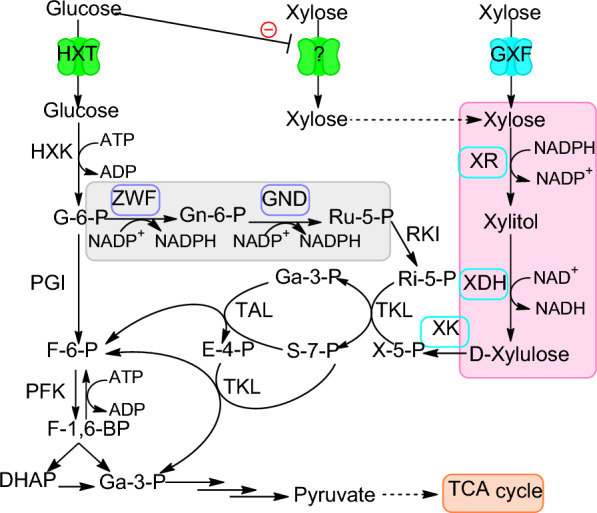
A schematic illustration of the strategies used in the current study to construct recombinant *Y. lipolytica* strains able to ferment xylose efficiently. The heterologous genes introduced are indicated in cyan box and the endogenous genes overexpressed are shown in purple box. *XR* xylose reductase, *XDH* xylitol dehydrogenase, *GXF* xylose facilitator, *XK* xylulokinase, *ZWF* phosphoglucose dehydrogenase, *GND1* 6-phosphoglucose dehydrogenase

**Fig. 4:**
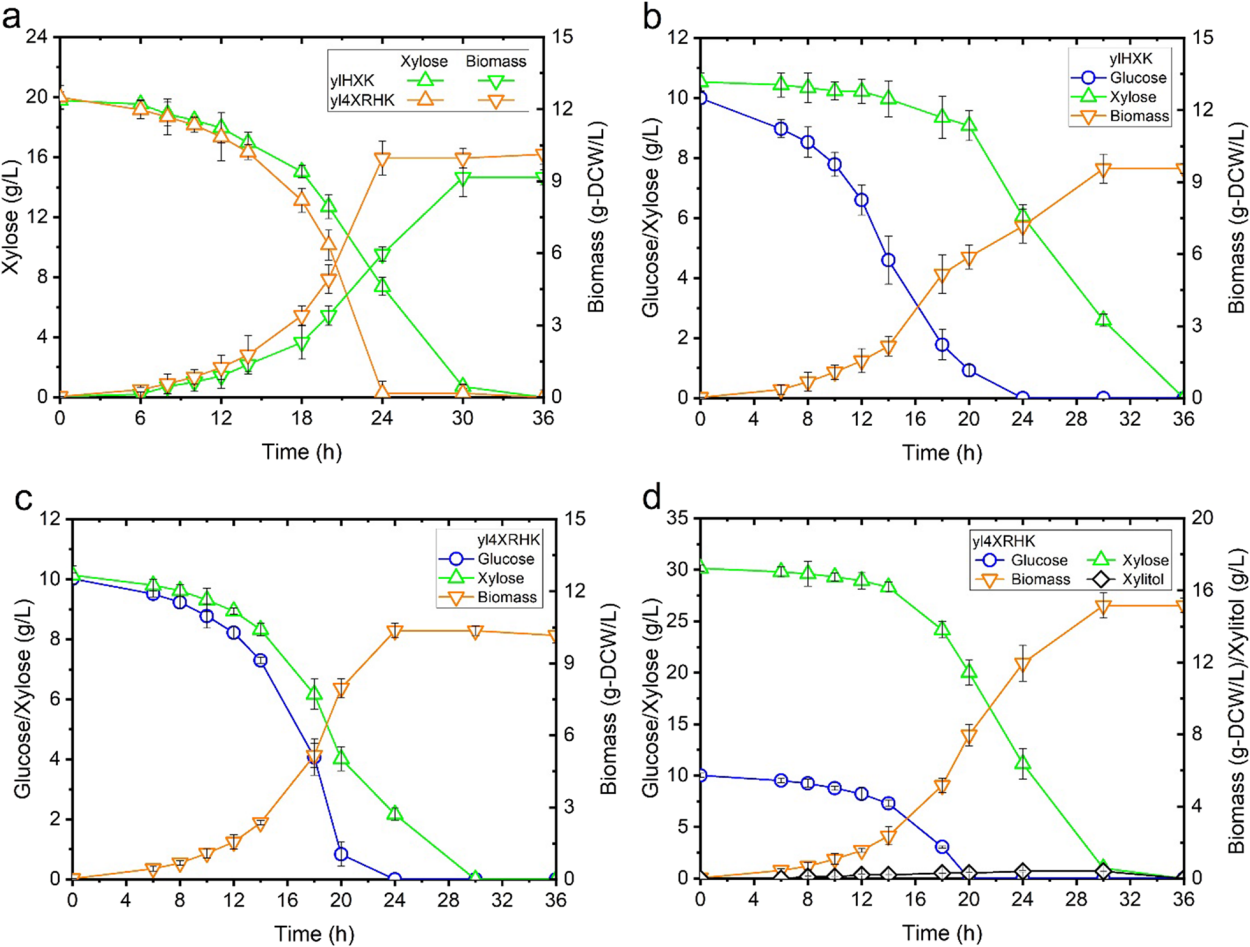
Comparsion of the growth of recombinant *Y. lipolytica* ylHXK (*pTEF-SsXR*, *pTEF-SsXDH*, *pTEF-XK*) and yl4XRHK (*p4UTef-SsXR, p4UTef-SsXDH, p4UTef-XK, pTEF-GiGXF1, pTEF-ZWF1, pTEF-GND1*) in areobic growth on (**a**) xylose; (**b**), (**c**) and (**d**) mixed glucose and xylose. Shown are biomass, xylose and glucose concentration versus time. Each data point represents the mean of three independent experiments and the error bar indicates the standard deviation

In this work, we first enhanced the expression of the three key genes *SsXR*, *SsXDH* and *YlXK* for xylose assimilation using 4UASTef promoter. In addition, *CiGXF1* was installed to facilitate xylose uptake and to bypass the glucose repression [21]. Moreover, the genes *ZWF1* and *GND1* of the pentose phosphate pathway, which may lead to increased cytoplasmic NADPH supply and enhanced xylose fermentation by intermediates feeding, were also overexpressed (Fig. 3) [22, 23]. Unlike *S. cerevisiae* for which the growth on xylose was always less efficient than glucose despites great endeavors made on pathway optimization [24, 25], our engineered strain yl4XRHK exhibited similar growth rate (0.18 h^−1^) and biomass yield (0.51 g-DCW/g-xylose) on xylose to that of obtained on glucose (Fig. 3, Table 1). Compared to the parental strain ylXHK, yl4XRHK exhibited a 6 h shorter fermentation time and a 30% higher specific xylose consumption rate. We also applied a high xylose and glucose ratio of 3:1 to investigate the impact of high glucose concentration on xylose consumption. Expressing *CiGXF1* greatly relieved the glucose repression on xylose fermentation, as simultaneous co-consumption of xylose and glucose was observed for the strain yl4XRHK [21]. For all the cultures, xylitol production was negligible even at high xylose/glucose ratio, for which 24 g/L of xylose remained to be consumed after glucose depletion (Fig. 4d).

### Development of recombinant strains of *Y. lipolytica* to co-ferment cellodextrins and xylose efficiently

**Fig. 5:**
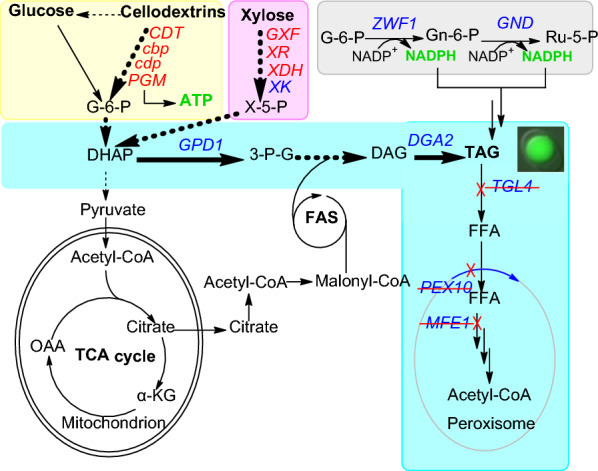
A schematic illustration of the strategies used in the current study to construct a cellodextrin- and xylose-fermenting *Y. lipolytica* for lipid overproduction. The pathway for cellodextrin phosphorolysis is marked with light yellow box, while xylose assimilation pathway is marked with pink box. Lipid pathway engineering is indicated in cyan box. PPP pathway overexpression to enhance NADPH supply is marked with gray box. The heterologous genes are written in red and the native genes are given in blue. The interrupted genes are marked with a red deletion line. *GPD1* glycerol-3-phosphate dehydrogenase, *DGA2* acyl-CoA:diacylglycerol acyl transferase, *MFE1* multifunctional beta-oxidation enzyme hydratase-dehydrogenase-epimerase, *PEX10* peroxisome biogenesis, *TGL* TAG lipase

Encouraged by the success of the above work, we then pursued a more ambitious goal to render *Y. lipolytica* with both xylose utilization and cellodextrins catabolism ability (Fig. 5). To achieve this, the genes for xylose utilization (*SsXR*, *SsXDH*, *YlXKS1* and *CiGXF1*) were introduced into the strain yl4BPT harboring cellodextrin-phosphorolysis pathway (*Ctcbp1*, *Ctcdp1*, *NcCDT1* and *ScPGM2*), resulting the strain yl4BX. Please note that the key genes *SsXR*, *SsXDH*, *ylXKS1*, *Ctcbp1*, *Ctcdp1* and *NcCDT1* were expressed under 4UASTef promoter, while the rest genes were controlled by the normal TEF promoter.

Thereafter, the strain yl4BX was characterized in YNB media containing different carbon source. The results showed that yl4BX required 26 h, 30 h, 32 h and 32 h to consume the single carbon source, glucose (20 g/L), xylose (20 g/L), cellobiose (19 g/L) and cellotriose (18.6 g/L), respectively (Table 2). Generally speaking, the lag phase for the cultures containing glucose was slightly shorter than those were deficient in glucose, and simultaneous co-consumption of these carbon sources was achieved. These results confirmed the positive effect of so-called ‘helper substrate’ to promote the use of less favourable substrate by nourishing the cells to achieve good fitness [14, 26]. As a result, the time needed for the complete depletion of carbon source including xylose (10 g/L)/cellobiose (9.5 g/L), xylose (10 g/L)/cellotriose (9.3 g/L) and xylose (10 g/L)/cellobiose (4.8 g/L)/cellotriose (4.7 g/L) was similar (32 h), but slightly longer than that of the fermentation on the mixture of xylose (10 g/L)/glucose (10 g/L), xylose (10 g/L)/glucose (5 g/L)/cellobiose (4.8 g/L), xylose (10 g/L)/glucose (5 g/L)/cellotriose (4.7 g/L). Strikingly, similar biomass yields (≈ 0.52 g/g-glucose equivalent in average) and growth rates (≈ 0.18 h^−1^ in average) were achieved for the above fermentations (Table 2). The production of xylitol was less than 1.0 g/L for all the xylose containing cultures.

**Table 2:** Comparison of the growth of recombinant strains of *Y. lipolytica* on xylose and glucose

Parameter	ylHXK		ylBPT		
Substrate (g/L)	10 Xyl	10 Xyl + 10 Glc	10 Xyl	10 Xyl + 10 Glc	10 Xyl + 30 Glc
q (g-s/g-DCW/h)	0.33 ± 0.03	0.31 ± 0.01	0.35 ± 0.03	0.36 ± 0.01	0.36 ± 0.02
μ_max_ (h^−1^)	0.16 ± 0.01	0.18 ± 0.02	0.18 ± 0.02	0.19 ± 0.01	0.19 ± 0.02
q (g-s/g-DCW/h)	0.33 ± 0.03	0.31 ± 0.01	0.35 ± 0.03	0.36 ± 0.01	0.36 ± 0.02
Fermentation time (h)	30	30	24	26	30
Residual substrate	0	0	0	0	0

± the standard deviation

### Engineering *Y. lipolytica* to accumulate high cellular content of lipids on LC biomass derived sugars

After the construction of a recombinant *Y. lipolytica* that is able to ferment xylose and cellodextrins in the presence of glucose efficiently, we then explored how his strain can be applied for lipids production on these carbon sources. The strategy we used to increase lipid accumulation is to overexpress *GPD1* and *DGA2*, which are involved in TAG formation, and to interrupt *MFE1*, *PEX10* and *TGL4* to prevent ß-oxidation, peroxisome biogenesis [27] and the release of fatty acids from the lipid body [28] (Fig. 5). The contribution of each single overexpression and gene deletion was verified in lipid production on glucose (Additional file 1: Fig. S1). The strain yl4BXP which exhibited the highest lipid accumulation yield on glucose (45% of the biomass) was inoculated into the media containing a mixture of xylose, cellobiose and cellotriose for further investigation. Lipid production was conducted in a 3L-bioreactor in the presence of 25 g/L of each sugar. A second addition of 25 g/L of each substrate was carried out when the total amount of carbon source dropped below 20 g/L. A C/N ratio of 60 was used according to the previous literature, which was reported as the best C/N ratio for lipid accumulation from different carbon sources [29].

**Fig. 6:**
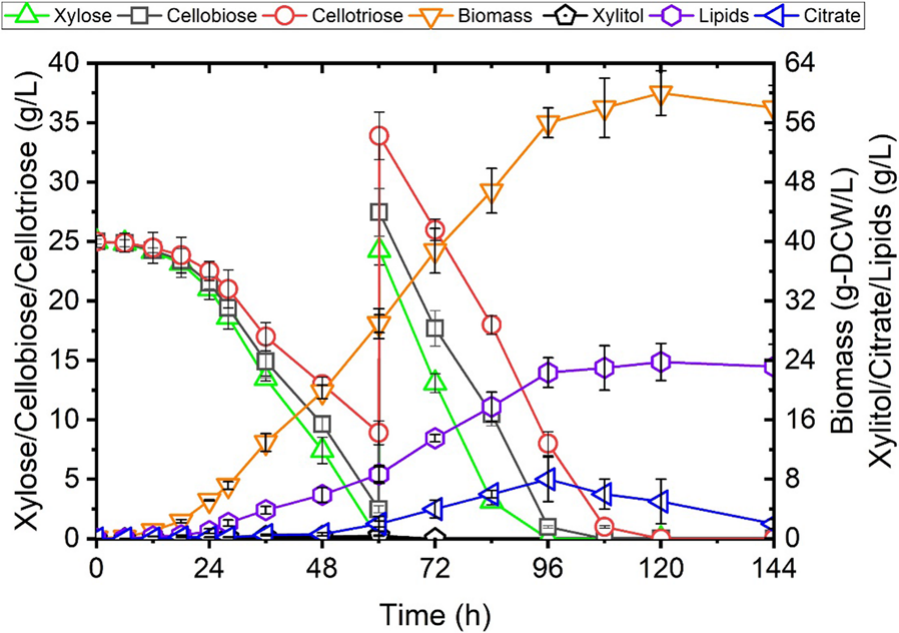
Lipid prodution of recombinant *Y. lipolytica* yl4BXP (*p4UTef-SsXR, p4UTef-SsXDH, p4UTef- XK, pTEF-GiGXF1, pTEF-ZWF1, pTEF-GND1, pTEF-GPD1, pTEF-DGA2*) from mixed carbon source including xylose, cellobiose and cellotriose in YNB meida. Shown are xylose, cellobiose, cellotriose, biomass, xylitol, citrate and lipids concentration versus time

**Fig. 7:**
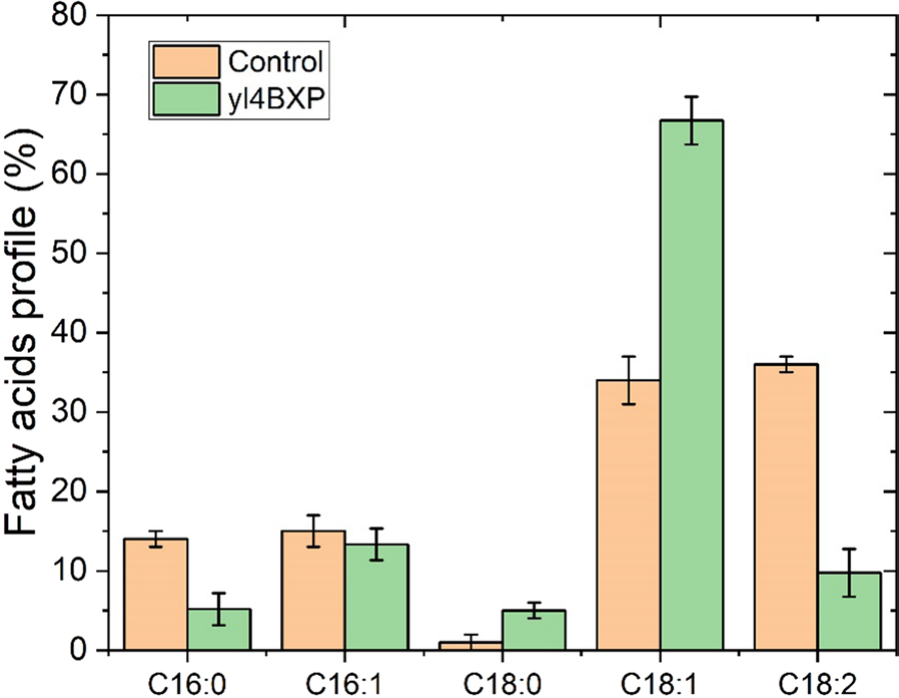
Comparison of fatty acid profile of recombinant *Y. lipolytica* yl4BXP (*p4UTef-SsXR, p4UTef-SsXDH, p4UTef- XK, pTEF-GiGXF1, pTEF-ZWF1, pTEF-GND1, pTEF-GPD1, pTEF-DGA2*) with the parental strain po1f-control in fed-batch SSF of steam-pretreated wheat straw in bioreactor

As illustrated in Fig. 6, co-consumption of the three sugars by yl4BXP was observed, with xylose being the fastest fermented sugar followed by cellobiose and cellotriose. In the end, yl4BXP consumed a total amount of 150 g/L mixed sugars in less than 5 days, and produced 59.6 g/L of biomass, a yield of 0.39 g-DCW/g-sugar. Lipid accumulation reached 23.8 g/L as estimated from the sum of the extracted fatty acids, a production of 0.16 g-lipid/g-sugar, corresponding to 40.0% of biomass (in DCW) and a productivity yield of 0.22 g/L/h (Fig. 6). Analysis of the fatty acid profile of yl4BXP illustrated a twofold increase in oleic acid (C18:1, 67%) and 73% decrease in linoleic acid (C18:2, 9.7%) in the engineered strain compared with the parental strain po1f-control. While linoleic acid (36%) and oleic acid (34%) was the first and second most abundant fatty acid in the parental strain, palmitoleic acid (C16:1), represents only 13% of total fatty acids, was the second most abundant fatty acid in yl4BXP (Fig. 7).

### Lipid production using recombinant *Y. lipolytica* in fed-batch SSF of wheat straw in bioreactors

To investigate whether the recombinant *Y. lipolytica* could be useful in the real scenario of LC biomass conversion, fed-batch SSF of steam-pretreated wheat straw was carried out in a 3L bioreactor using the strain yl4BXP. A sequential co-fermentation scheme was employed comprising an initial phase of batch culture on hydrolysate liquor followed by three additions of the cellulase and unwashed solids of the wheat straw slurry. It has been suggested that such a fermentation strategy is advantageous due to the detoxification of the inhibitory compounds by yeast during the batch phase and the improved tolerance of cells through progressive adaptation [30].

**Fig. 8:**
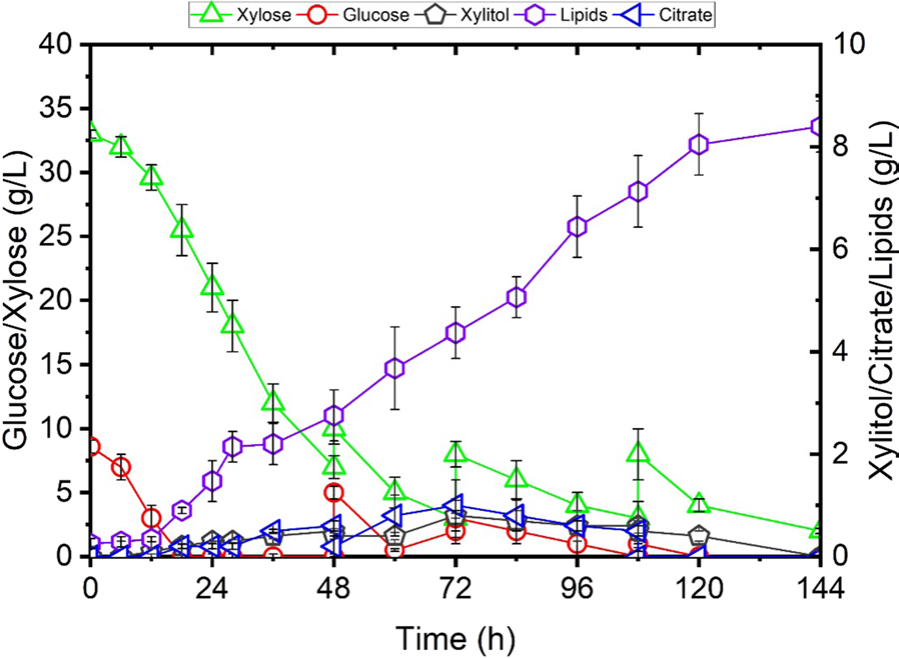
Lipid prodution of recombinant *Y. lipolytica* yl4BXP (*p4UTef-SsXR, p4UTef-SsXDH, p4UTef- XK, pTEF-GiGXF1, pTEF-ZWF1, pTEF-GND1, pTEF-GPD1, pTEF-DGA2*) by fed-batch SSF of steam-pretreated wheat straw in bioreactor. Shown are glucose, xylose, biomass, xylitol, citrate and lipid concentration versus time

During the initial 48 h of batch cultivation, 8.6 g/L glucose was depleted and 26 g/L of xylose representing 79 wt% of the total available xylose in hydrolysate was consumed, and 2.76 g/L of lipids were produced (Fig. 8). To evaluate the feasibility of the process, Cellic CTec2 was added at a dosage to avoid excessive hydrolysis of glucan into glucose (10.0 FPU/g cellulose), and also at a higher dosage of 15.0 FPU/g cellulose for comparison. During the subsequent fed-batch SSF at lower enzyme dosage, xylose was co-consumed with the released sugars from enzymatic hydrolysis of glucan. No accumulation of glucose and cellodextrins was observed, and this observation was correlated with lipid production (detected in the form of FAMEs), reflecting a continuous biomass formation. After 144 h of cultivation, the fed-batch SSF resulted in a lipid concentration of 8.5 g/L, which corresponded to an overall production yield of 0.1 g-lipid/g-sugar (Fig. 8). Glucose was depleted, and 2.3 g/L of xylose sustained in the end of fermentation. Xylose utilization reached 96 wt% of the total xylose loading. The co-consumption of glucose, xylose and cellodextrins were sustained throughout the 144 h of co-fermentation. Prolonging the fermentation did not yield further gain in lipid titer.

## Discussions

It is interesting to note that phosphorolytic capability was recently conferred to *S. cerevisiae* (expressing *Nc*Cdt1 and a Cbp from *Saccharomyces degradans*) [18]. In the previous work, the energetic benefits of phosphorolysis were evidenced because *S. cerevisiae* endowed with the cellobiose phosphorolysis pathway produced more biomass and ethanol than a cellobiose-hydrolyzing strain. Consistent with these observations, the newly developed strain in this work showed better performance, e.g., shorter lag phase, faster rates of growth and substrate consumption, than the previously engineered yeast relied on cellobio-hydrolytic activity [31]. However, our results imply that the presence of the phosphorolysis pathway in *Y. lipolytica* did not procure an obvious energetic benefit, as the biomass yield of yl4BPT was similar to the previous reported *Y. lipolytica* possessing cellobiose hydrolytic activity [31]. It is noteworthy that further overexpressing either *Ctcbp*, *Ctcdp* or *CDT1* using 8UASTef promoter did not improve the growth rate and biomass yield further. Nevertheless, yl4BPT is still the most efficient cellodextrin-fermenting recombinant yeast reported so far.

Several recent studies have shared the same interest in developing a xylose-fermenting *Y. lipolytica* by employing different strategies. As such, although *Y. lipolytica* PO1g overexpressing *SsXR* and *SsXDH* was unable to grow on xylose, the following adaptation has enabled the growth of the strain on xylose with a doubling time of 25 h [26]. In a second work, *Y. lipolytica* YlSR001 was directly adapted in xylose culture which allowed the isolation of a mutant strain capable of slightly growing on xylose [32]. The author disclosed that the insufficient XDH activity was the limiting factor for xylose assimilation. The overexpression of XDH enhanced the growth of the strain on xylose, but unfortunately, still at an extremely slow rate [32]. Although our previous strategy to construct a xylose-fermenting *Y. lipolytica* by overexpressing *SsXR*, *SsXDH* and *YlXK* was successful, xylose assimilation in the presence of glucose of the engineered strain was not optimal as sequential consumption of xylose after glucose was still the case due to glucose repression [14]. In addition, the accumulation of xylitol on xylose occurred after glucose depletion when a high initial concentration of xylose was applied, which indicated *Y. lipolytica* suffered from the cofactor imbalance issue since *ssXR* consumes NADPH and XDH generates NADH [26]. In this respect, our current strategy was obviously more successful. CiGxf1p is a non-glucose preference transporter which shows highest efficacy in xylose uptake in the presence of glucose [21]. Its V_max_ value is one order of magnitude higher than the xylose-H^+^ symporter CiGxs1p [33], and the overexpression of CiGxf1p in *Kluyveromyces marxianus* has greatly improved xylitol production due to the enhanced xylose uptake [34]. Consistent with previous study [34], the installation of CiGxf1p in *Y. lipolytica* facilitated xylose uptake and relieved the glucose repression. Considering the presence of significant amount of glucose is prevailing in real LC biomass hydrolysates, eliminating glucose repression on xylose utilization holds great advantage. In addition, it has been demonstrated that Zwf1p and Gnd1p in the PPP play an essential role in cytoplasmic NADPH generation in *Y. lipolytica* [35], and their overexpression promoted NADPH regeneration in *Y. lipolytica* which led to increased erythritol [36] and scutellarin production [23]. Similarly, overexpressing the genes *ZWF1* and *GND1* led to increased cytoplasmic NADPH supply enhanced xylose fermentation by maintaining cofactor balance. Moreover, overexpression of Zwf1p and Gnd1p enhanced the metabolic flux of the PPP which may contribute to better growth and lipid production [22, 23]. Surplus NADPH and enhanced xylose catabolism support active mitochondrial function under aerobic conditions which may in turn contribute to a better NADPH/NADH equilibrium [37]. The integration of both xylose and cellodextrin assimilation pathways did not provoke unknown remodulations of the metabolism of the engineered strain in terms of redox balance.

In recent work, attempt to produce lipid from xylose has yielded a modified strain which produced 20 g/L of lipid from 150 g/L of xylose, a lipid accumulation of 35% of DCW [14]. Under high C/N ratio condition, xylose remained unused in the end of the culture due to the imbalanced C/N ratio and the lack of necessary nutrition. In contrast to the production of high concentrations of xylitol (~ 9.0 g/L) and citric acid (~ 25.0 g/L) in the previous work [14], the production of xylitol and citric acid under our condition was not significant (Fig. 6). This is likely due to the overexpression of the genes *ZWF1* and *GND1* which contributed to a better redox balance in yl4BXP. Analysis of the fatty acid profile revealed that yl4BXP contained 80% of monounsaturated fatty acids, 39% higher than that of the control. This was mainly due to the increased level of oleic acid (C18:1) and decreased level of linoleic acid (C18:2) in yl4BXP. It has been suggested that monounsaturated fatty acids are more favourable for biodiesel production as they demonstrated better oxidation stability than polyunsaturated fatty acids [38]. Therefore, the strain yl4BXP developed here holds great potential for the production of fatty acid prosecutors for biodiesel application. For lipid production in fed-batch SSF of wheat straw hydrolysate, the low lipid titer was mainly due to the limited biomass production yield (25 g-DCW/L), even though moderate lipid accumulation was achieved (34%). Since similar fermentation profile was obtained when a higher cellulase loading was applied, the unavailability of carbon source in the culture medium was not the limiting factor. The low biomass yield was probably due to the presence of inhibitory compounds, such as acetic acid (> 5 g/L) and furfural (> 3 g/L) (Additional file 1: Figure S4), at the level for which has been shown to limit the growth of the yeast in wheat straw hydrolysate fermentation [39].

In summary, we confirm the development of the first xylose/cellodextrin fermenting obese yeast yl4BXP, which is able to accumulate high cellular content of lipids on xylose/cellodextrins. To our knowledge, this is the first study to demonstrate how *Y. lipolytica* can be metabolically engineered to co-ferment cellodextrins and xylose to produce lipids from enzymatic hydrolysate of wheat straw at lower enzyme loading, and thus lowering the production cost. Therefore, the modified strain of *Y. lipolytica* developed here holds great potential for the production of industrial relevant compounds through cost-effective process.

## Conclusions

This study clearly demonstrated that *Y. lipolytica* can be metabolically engineered to both efficiently consume the non-native substrates xylose and cellodextrins and to convert them into lipids. No accumulation of reducing sugars were observed which suggests the engineered strain satisfied the requirements for β-glucosidase activity. Apparently, incorporation of the cellodextrin-phosphorololytic *Y. lipolytica* into a SSF process is beneficial as reduced loading of external cellulases is possible, and thus lowering the overall process cost. However, lipid production on hydrolysate was still not comparable to that on defined media in terms of titer, yield and productivity, probably due to the impact of inhibitors of the hydrolysate. This also constitutes an attractive target for strain engineering in the future, aiming to develop more robust strains that can tolerate high levels of inhibitory compounds in hydrolysate. Nevertheless, this is the first step towards cost-effective production of valuable chemicals using engineered *Y. lipolytica* from lignocellulosic biomass.

## Methods

### Strains and culture media

The microbial strains used in this work are summarized in Table 3. *E. coli* DH5 was used for plasmid propagation and construction. *Y. lipoltyica* Po1f [40] was routinely cultivated in a medium containing 10 g/L yeast extract, 20 g/L peptone and 20 g/L glucose (YPD). Transformant selection was performed on solid YNB medium containing 1.7 g/L YNB, 10 g/L glucose, xylose, cellobiose or cellotriose, and 5 g/L ammonium chloride. Leucine or uracil was added at the concentration of 440 mg/L according to the auxotrophic requirement. The YNB medium for lipid production contained 25 g/L xylose, 25 g/L cellobiose and 25 g/L cellotriose, with a second feeding of each sugar when the concentration of the total reducing sugars drop bellowed 20 g/L. NH_4_Cl was added into the media at the concentration to yield a C/N ratio of 60:1.

**Table 3:** Microbial strains used in the present study

Strains	Relevant genotype	Source of reference
*E. coli* DH5α	Φ80dlacZΔm15, *recA1*, *endA1*, *gyrA96*, *thi-1*, *hsdR17* (rk^−^, mk^+^), *supE44*, *relA1*, *deoR*, Δ(*lacZY*A-argF) U169	Invitrogen
*Y. lipolytica* Po1f	*MatA*, *leu2-270*, *ura3-302*, *xpr2-322 axp1*	Madzak et al., 2000
po1f-1	Po1f; Δ*MEF1,* Δ*PEX10,* Δ*TGL4*	This investigation
po1f-control	Po1f; *URA3*, *LEU2*	This investigation
ylP	Po1f; *pTEF-Ctcdp*	This investigation
ylPT	Po1f; *pTEF-NcCDT1*, *pTEF-Ctcdp*	This investigation
ylBPT	Po1f; *pTEF-NcCDT1, pTEF-Ctcdp, pTEF-Ctcdp, pTEF-ScPGM2*	This investigation
yl4BPT	Po1f; *p4UTef-NcCDT1, p4UTef-Ctcdp, p4UTef-Ctcdp, pTEF-ScPGM2*	This investigation
yl8BPT	Po1f; *p8UTef-NcCDT1, p8UTef-Ctcdp, p8UTef-Ctcdp, pTEF-ScPGM2*	This investigation
*yl*XHK	Po1f; *pTEF-SsXR, pTEF-SsXDH, pTEF-XK*	This investigation
yl4XRHK	Po1f; *p4UTef-SsXR, p4UTef-SsXDH, p4UTef-XK, pTEF-GiGXF1, pTEF-ZWF1, pTEF-GND1*	This investigation
yl4BX	yl4BPT; *p4UTef-SsXR, p4UTef-SsXDH, p4UTef- XK, pTEF-GiGXF1, pTEF-ZWF1, pTEF-GND1*	This investigation
yl4BXP	po1f-1; yl4BPT; *p4UTef-SsXR, p4UTef-SsXDH, p4UTef- XK, pTEF-GiGXF1, pTEF-ZWF1, pTEF-GND1, pTEF-GPD1, pTEF-DGA2*	This investigation

Lipid production on wheat straw hydrolysates was carried in defined media containing NH_4_Cl (at a C/N ratio of 60:1) and supplemented with trace elements, vitamins and salts according to the previous report [41].

### Plasmid construction

The plasmids constructed for gene expression in the present study are summarized in Additional file 1: Table S1. The primers used for PCR are listed in Additional file 1: Table S2. First of all, the plasmids pYL1/pYU1, pYL4/pYU4 and pYL8/pYU8, derived from the plasmid pYLXP [42], were constructed. The plasmid pYL1 contains a *LEU2* selection marker flanking with a *loxP* site and a 500-bp sequence on each end, one of which is homologous to the upstream and the other to the downstream of *URA3* gene. Similarly, pYU1 contains a *URA3* selection marker flanking with a *loxP* site and a 500-bp sequence on each end, one of which is homologous to the upstream and the other to the downstream of *LUE2* gene. Replacing the *TEF* promoter of the above plasmids by 4UASTef and 8UASTef promoter [43] yield the plasmids pYL4/pYU4 and pYL8/pYU8.

To construct the plasmid for cellodextrin utilization, the DNA sequences of *Ctcbp* (GenBank accession number: AB013109.1), *Ctcdp* (GenBank accession number: AB006822.1) and *NcCDT1* (GenBank accession number: NC_026501.1) were synthesized by Tianlin Biotech (Wuxi, China), introducing optimal codon usage features for *Y. lipolytica*, and directly cloned into the *Xba*I/*Kpn*I sites of the plasmids pYL1 and pYL4, to generate the plasmids pYL1-Ctcbp1, pYL1-Ctcdp1, pYL1-NcCDT1, pYL1-ScPGM2, pYL4-Ctcbp1, pYL4-Ctcdp1, pYL4-NcCDT1 and pYL4-ScPGM2. The gene *ScPGM2* (GenBank accession number: NM_001182605.1) was amplified from the gDNA of *S. cerevisiae* and cloned into the *Xba*I/*Spe*I sites of the plasmids pYL1 and pYL4, to generate the plasmids pYL1-ScPGM2 and pYL4-ScPGM2. And then, the expression cassettes containing promoter-target gene-*XPR2* terminator were amplified from the above plasmids and were subsequently assembled with the PCR fragment of plasmid pYL1, generating the co-expression plasmids pYL1-BDP and pYL4-BDP. The primers for DNA assembly contained a ∼15 bp homologous region to the neighborhood fragments and/or the plasmid backbone on its end (Table 4).

**Table 4:** Comparison of the growth of recombinant strains of *Y. lipolytica* on xylose, glucose and cellodextrins

Carbon source	Lag phase (h)	μ_max_ (h^−1^)	q (g-s/g-DCW/h)	Biomass yield (g-DCW/ s-g)	Fermentation time (h)
G_1_(20 g/L)	4	0.20 ± 0.01	0.35 ± 0.03	0.54 ± 0.01	26
X1(20 g/L)	6	0.18 ± 0.00	0.33 ± 0.04	0.51 ± 0.02	30
G_2_(19 g/L)	8	0.17 ± 0.02	0.32 ± 0.02	0.52 ± 0.00	32
G_3_(18.6 g/L)	8	0.18 ± 0.02	0.32 ± 0.03	0.55 ± 0.03	32
Xl(10 g/L)/G_2_(9.5 g/L)	6	0.18 ± 0.01	0.33 ± 0.01	0.53 ± 0.01	32
Xl(10 g/L)/G_3_(9.3 g/L)	4	0.20 ± 0.02	0.35 ± 0.02	0.54 ± 0.03	26
Xl(10 g/L)/G_2_(4.8 g/L)/C_3_(4.7 g/L)	6	0.18 ± 0.01	0.33 ± 0.00	0.51 ± 0.02	30
Xl(10 g/L)/G_1_(10 g/L)	8	0.17 ± 0.00	0.32 ± 0.02	0.52 ± 0.03	32
X1(10 g/L)/G_1_(5 g/L)/G_2_(4.8 g/L)	8	0.18 ± 0.02	0.32 ± 0.01	0.55 ± 0.01	32
X1(10 g/L)/G_1_(5 g/L)/G_3_(4.7 g/L)	6	0.18 ± 0.01	0.33 ± 0.02	0.53 ± 0.03	32

± the standard deviation. The concentration of cellobiose or cellotriose in culture media was equivalent to that of 5 or 10 g/L glucose after hydrolysis

Similarly, to construct the plasmid for xylose assimilation, the gene *XR* encoding xylose reductase (GenBank accession number: XM_001385144.1), the gene *XDH* encoding xylitol dehydrogenase (GenBank accession number: XM_001386945.1), was amplified from the gDNA of *S. stipitis* and cloned into *Xba*I/*Kpn*I and *Xba*I/*Spe*I site of the plasmid pYL1 or pYL4, to generate the plasmids pYU1-SsXR, pYU1-SsXDH, pYU4-SsXR and pYU4-SsXDH. The gene *GXF1* (GenBank accession number: AJ937350.1) was codon optimized and synthesized, and directly cloned into the plasmids pYL1 and pYL4, to generate the plasmids pYU1-CiGXF and pYU4-CiGXF, respectively. The gene *XKS1*, encoding *Y. lipolytica* xylulokinase (YALI0F10923g), and the gene *ZWF1*, encoding glucose-6-phosphate dehydrogenase and the gene *GND1* (YALI0E22649g), encoding 6-phosphogluconate dehydrogenase (YALI0B15598g), was amplified from gDNA of *Y. lipolytica* by PCR and cloned into the *Xba*I/*Kpn*I sites of the plasmids pYU1 and pYU4, to generate the plasmids pYU1-XKS, pYU1-ZWF, pYU1-GND, pYU4-XKS, pYU4-ZWF and pYU4-GND, respectively. And then, the expression cassettes containing promoter-target gene-*XPR2* terminator were amplified from the above constructed plasmids and were subsequently assembled with the PCR fragment of plasmid pYL1, generating the co-expression plasmids pYU1-XRK and pYU4-XRK. The primers for DNA assembly contained a ∼15 bp homologous region to the neighborhood fragments and/or to the plasmid backbone on its end.

To construct the plasmid for lipid overproduction, the gene *GPD1* (YALI0B02948g), encoding glycerol-3-phosphate dehydrogenase, and the gene *DGA2* (YALI0D07986g), encoding acyl-CoA:diacylglycerol acyl transferase, was amplified from gDNA of *Y. lipolytica* by PCR and cloned into the plasmids pYU1 and pYU4, to generate the plasmids pYU1-GPD and pYL1-DGA, respectively. The success in the construction of the desired plasmid was confirmed by DNA sequencing.

### Gene deletion

The gene *MFE1* (YALI0E15378g) encoding multifunctional beta-oxidation enzyme hydratase–dehydrogenase–epimerase involved in beta-oxidation [44], the gene *PEX10* (YALI0C01023g) involved in peroxisome biogenesis [45], and the gene *TGL4* (YALI0F10010g) encoding an lipase to degrade TAG [46], was deleted from the genome of *Y. lipolytica* Po1f using CRISPR/Cas9 technology following the instructions of previous study [47]. The single guide RNA (sgRNA) used in this work is listed in Additional file 1: Table S3. The successful deletion of the target genes of *Y. lipolytica* was verified by PCR followed by DNA sequencing.

### Strain transformation and selection

For yeast transformation, the plasmids were linearized and introduced into *Y. lipolytica* po1f and po1f-1 (Δ*MEF1*, Δ*PEX10*, Δ*TGL4*) using the Frozen-EZ Yeast Transformation II Kit (Zymo Research, USA). Transformant selection was performed on YNB plate containing the specific carbon source according to the integrated pathway and the auxotrophic genotype. The *loxP*-Cre recombination system was used for marker reuse during multistep insertion of the target genes [42]. The successful incorporation of multiple genes into *Y. lipolytica*’s genome was verified by PCR using the gene specific primers. Ten transformants of each construct were grown in liquid YNB media supplemented with the suitable carbon source (xylose, cellobiose or cellotriose) and the transformant showed an average growth profile was selected for the further analysis.

### Measurement of enzyme activity

Cellobiose and cellodextrin phosphorylase activities were measured by detecting Glc-1P generated from cellobiose and cellotriose according to the previous report [48]. One unit of activity (U) was defined as the amount of enzyme required to release 1 μmol Glc-1P per min.

### Determination of intercellular metabolites

The cells were recovered from the culture media by centrifugation (8000 × g for 5 min at 4 °C). Cellular Glc-1P of the crude cell extract was measured using an enzymatic kit (G1P Colorimetric Assay Kit, Sigma). Cellular concentrations of cellobiose and glycogen were determined using the method described before [49]. Free glucose was measured using the D-Glucose Assay Kit.

### Lipid production on sugars derived from LC biomass in bioreactor

The preculture was carried out in YNB media and used to inoculate 1.2L YNB media containing xylose (25 g/L), cellobiose (25 g/L) and cellotriose (25 g/L) in a 3.0-L stirred-tank bioreactor (Sartorius, Germany) to reach an initial OD600nm of 1.0. A feeding of 25 g/L of each sugar was performed when the concentration of the total reducing sugars dropped below 20 g/L. Throughout the fermentation process, the pH was maintained at 5.5 with the automatic addition of 2.0 M NaOH and the temperature was kept at 28 °C. An aeration of 0.5 vvm was set up and the stirring speed was automatically controlled to keep the dissolved oxygen at 20% of air saturation. Samples were taken regularly to analyze the concentrations of biomass, metabolites and carbon source in culture media.

### Wheat straw processing

Diluted acid-catalyzed steam treatment of wheat straw was carried out as described previously [39]. Briefly, the wheat straw was impregnated with 0.5% H_2_SO_4_ at pH 1.7, and then incubated at 187 °C for 8 min in an autoclave. The water-insoluble solids (WIS) content of the pretreated wheat straw was about 12 wt%. The composition of steam-pretreated wheat straw is given in Additional file 1: Table S4.

### Lipid production on fed-batch SSF of steam-pretreated wheat straw in bioreactor

To reduce the impact of inhibitors on SSF of *Y. lipolytica* and to improve the substrate conversion yield, a sequential fermentation strategy was employed, which is comprising a batch fermentation of the xylose/glucose-rich hydrolysate liquor followed by fed-batch SSF of solid wheat straw slurry. Yeasts were pre-cultivated in defined media and then used to inoculate 1.2 L hydrolysate in a 3 L stirred-tank bioreactor (Sartorius, Germany) to yield an initial biomass concentration of 2.5 g-DCW/L. During the fermentation process, the pH was constantly maintained at 5.0 and the temperature was kept at 30 °C. An aeration of 0.5 vvm and a dissolved oxygen of 20% of air saturation was sustained. Three arounds feeding of 40 g/L of solid wheat straw slurry into the bioreactor was carried out when the concentration of reducing sugars dropped below 10 g/L. This corresponding to a total WIS load of 12 wt%. Cellic CTec2 was added at a load of 10 FPU g/WIS. The fermentation was pursued for 6 days and samples were taken at regular intervals for further analysis.

### Analysis of substrate consumption and product and biomass formation

The concentration of glucose, xylose, cellobiose, cellotriose, xylitol and citric acid in the culture supernatant was analyzed by HPLC equipped with an aminex HPX87-H column as described previously [14]. Detection of glucose, xylose, xylitol, cellobiose and cellotriose was realized by a Shodex RI-101 refractive index detector, while citric acid was detected by an UV detector (210 nm).

To determinate dry cell weight (DCW), cell pellets were recovered from the culture media and then filtrated, washed, dried and weighed.

Lipid quantification was carried out according to the protocols described before [31]. Briefly, C17:0 (Sigma) (50 μg) was added as the internal standard, and lipids were extracted from freeze-dried cells (∼10 mg). After methylation, the fatty acid methyl esters (FAMEs) were measured by gas chromatography (8891 GC System, Agilent, USA) equipped with a HP-5 GC column (30 m × 0.32 mm × 0.5 μm, Agilent, USA). A split mode of 1 μL at 250 °C was employed with helium as the carrier gas (2 mL/min). The temperature program was ramping from 120 °C to 250 °C in 20 min by three steps (10 °C/min for 6 min, 0.33 °C/min for 9 min and 15 °C/min for 5 min). FAMEs were detected by a flame ionization detector (FID) at 270 °C (2.0 pA) and quantified by comparing with the standards of known concentration.

### Calculations and statistics

Specific rate of substrate consumption (g-s/g-DCW/h) was estimated according to the following equation:${\mathrm{q}}_{\text{sub}}=\frac{{\mu }_{\mathrm{max}}}{{Y}_{\text{X/s}}}$, where ${Y}_{\text{X/s}}=\frac{\text{dX}}{{\text{ds}}}$ represents the biomass yield coefficient. The biomass and product yields were calculated as the ratio of the amount of biomass or products formed divided by the amount of carbon source consumed. The maximum specific growth rate *μ*_max_ (h^−1^) was calculated from the plot of biomass concentration versus time for exponentially growing cells. The duration of lag phase was estimated using an online tool as described previously [50]. All the experiments were performed at least in triplicate, and the mean values ± standard deviation were shown.

### Supplementary Information

**Additional file 1**: **Table S1** Plasmids used or created in the present study. **Table S2** The sequences of the oligonucleotide primers used in this study. **Table S3** sgRNA for gene deletion. **Table S4** The composition of steam-pretreated wheat straw.

## Data Availability

All data generated or analyzed in the present study are included in this published article and a supporting material “Additional file 1”.

## References

[1] Raj T, Chandrasekhar K, Naresh Kumar A, Rajesh Banu J, Yoon JJ, Kant Bhatia S, Yang YH, Varjani S, Kim SH (2022). Recent advances in commercial biorefineries for lignocellulosic ethanol production: Current status, challenges and future perspectives. Bioresour Technol.

[2] Stockton BC, Mitchell DJ, Grohmann K, Himmel ME (1991). Optimumβ-D-glucosidase supplementation of cellulase for efficient conversion of cellulose to glucose. Biotech Lett.

[3] Cunha JT, Soares PO, Romaní A, Thevelein JM, Domingues L (2019). Xylose fermentation efficiency of industrial Saccharomyces cerevisiae yeast with separate or combined xylose reductase/xylitol dehydrogenase and xylose isomerase pathways. Biotechnol Biofuels.

[4] Peña-Castro JM, Muñoz-Páez KM, Robledo-Narvaez PN, Vázquez-Núñez E (2023). Engineering the metabolic landscape of microorganisms for lignocellulosic conversion. Microorganisms.

[5] Kao MR, Yu SM, Ho TUD (2021). Improvements of the productivity and saccharification efficiency of the cellulolytic β-glucosidase D2-BGL in Pichia pastoris via directed evolution. Biotechnol Biofuels.

[6] You S, Li J, Zhang F, Bai Z-Y, Shittu S, Herman R-A, Zhang W-X, Wang J (2021). Loop engineering of a thermostable GH10 xylanase to improve low-temperature catalytic performance for better synergistic biomass-degrading abilities. Biores Technol.

[7] Moreno AD, Carbone A, Pavone R, Olsson L, Geijer C (2019). Evolutionary engineered Candida intermedia exhibits improved xylose utilization and robustness to lignocellulose-derived inhibitors and ethanol. Appl Microbiol Biotechnol.

[8] Vu VNH, Kohári-Farkas C, Filep R, Laszlovszky G, Ban MT, Bujna E, Gupta VK, Nguyen QD (2023). Design and construction of artificial microbial consortia to enhance lignocellulosic biomass degradation. Biofuel Research Journal.

[9] Groenewald M, Boekhout T, Neuvéglise C, Gaillardin C, van Dijck PW, Wyss M (2014). *yarrowia lipolytica*: safety assessment of an oleaginous yeast with a great industrial potential. Crit Rev Microbiol.

[10] Beopoulos A, Chardot T, Nicaud JM (2009). *Yarrowia lipolytica*: a model and a tool to understand the mechanisms implicated in lipid accumulation. Biochimie.

[11] Guo Q, Peng QQ, Chen YY, Song P, Ji XJ, Huang H, Shi TQ (2022). High-yield α-humulene production in *Yarrowia lipolytica* from waste cooking oil based on transcriptome analysis and metabolic engineering. Microb Cell Fact.

[12] Gallego-García M, Moreno AD, González A, Negro MJ (2023). Efficient use of discarded vegetal residues as cost-effective feedstocks for microbial oil production. Biotechnol Biofuels Bioprod.

[13] Wang J, Yu X, Wang K, Lin L, Liu HH, Ledesma-Amaro R, Ji XJ (2023). Reprogramming the fatty acid metabolism of *Yarrowia lipolytica* to produce the customized omega-6 polyunsaturated fatty acids. Bioresour Technol.

[14] Ledesma-Amaro R, Lazar Z, Rakicka M, Guo Z, Fouchard F, Coq AC, Nicaud JM (2016). Metabolic engineering of *Yarrowia lipolytica* to produce chemicals and fuels from xylose. Metab Eng.

[15] Guo ZP, Borsenberger V, Croux C, Duquesne S, Truan G, Marty A, Bordes F (2020). An artificial chromosome ylAC enables efficient assembly of multiple genes in *Yarrowia lipolytica* for biomanufacturing. Commun Biol.

[16] Galazka JM, Tian C, Beeson WT, Martinez B, Glass NL, Cate JH (2010). Cellodextrin transport in yeast for improved biofuel production. Science.

[17] Nakai H, Hachem MA, Petersen BO, Westphal Y, Mannerstedt K, Baumann MJ, Dilokpimol A, Schols HA, Duus J, Svensson B (2010). Efficient chemoenzymatic oligosaccharide synthesis by reverse phosphorolysis using cellobiose phosphorylase and cellodextrin phosphorylase from *Clostridium* thermocellum. Biochimie.

[18] Ha SJ, Galazka JM, Joong OhE, Kordić V, Kim H, Jin YS, Cate JH (2013). Energetic benefits and rapid cellobiose fermentation by *Saccharomyces* cerevisiae expressing cellobiose phosphorylase and mutant cellodextrin transporters. Metab Eng.

[19] Dulermo T, Nicaud JM (2011). Involvement of the G3P shuttle and beta-oxidation pathway in the control of TAG synthesis and lipid accumulation in *Yarrowia lipolytica*. Metab Eng.

[20] Beopoulos A, Haddouche R, Kabran P, Dulermo T, Chardot T, Nicaud JM (2012). Identification and characterization of DGA2, an acyltransferase of the DGAT1 acyl-CoA:diacylglycerol acyltransferase family in the oleaginous yeast *Yarrowia lipolytica*. New insights into the storage lipid metabolism of oleaginous yeasts. Appl Microbiol Biotechnol.

[21] Runquist D, Hahn-Hägerdal B, Rådström P (2010). Comparison of heterologous xylose transporters in recombinant *Saccharomyces* cerevisiae. Biotechnol Biofuels.

[22] Qiao K, Wasylenko TM, Zhou K, Xu P, Stephanopoulos G (2017). Lipid production in *Yarrowia lipolytica* is maximized by engineering cytosolic redox metabolism. Nat Biotechnol.

[23] Zhang P, Wei W, Shang Y, Ye BC (2023). Metabolic engineering of *Yarrowia lipolytica* for high-level production of scutellarin. Bioresour Technol.

[24] Subtil T, Boles E (2012). Competition between pentoses and glucose during uptake and catabolism in recombinant *Saccharomyces* cerevisiae. Biotechnol Biofuels.

[25] Kim SR, Park YC, Jin YS, Seo JH (2013). Strain engineering of *Saccharomyces* cerevisiae for enhanced xylose metabolism. Biotechnol Adv.

[26] Stephanopoulos G, Tai M: Engineered microbes and methods for microbial oil overproduction from cellulosic materials. WO 2013/192520 A1 2013.

[27] Blazeck J, Hill A, Liu L, Knight R, Miller J, Pan A, Otoupal P, Alper HS (2014). Harnessing *Yarrowia lipolytica* lipogenesis to create a platform for lipid and biofuel production. Nat Commun.

[28] Dulermo T, Treton B, Beopoulos A, Kabran Gnankon AP, Haddouche R, Nicaud JM (2013). Characterization of the two intracellular lipases of Y lipolytica encoded by TGL3 and TGL4 genes: new insights into the role of intracellular lipases and lipid body organisation. Biochim Biophys Acta.

[29] Lazar Z, Dulermo T, Neuveglise C, Crutz-Le Coq AM, Nicaud JM (2014). Hexokinase-A limiting factor in lipid production from fructose in *Yarrowia lipolytica*. Metab Eng.

[30] Nielsen F, Zacchi G, Galbe M, Wallberg O (2016). Prefermentation improves ethanol yield in separate hydrolysis and cofermentation of steam-pretreated wheat straw. Sustainable Chem Processes.

[31] Guo Z, Duquesne S, Bozonnet S, Cioci G, Nicaud JM, Marty A, O'Donohue MJ (2015). Development of cellobiose-degrading ability in *Yarrowia lipolytica* strain by overexpression of endogenous genes. Biotechnol Biofuels.

[32] Ryu S, Hipp J, Trinh CT (2015). Activating and elucidating complex sugar metabolism in *yarrowia lipolytica*. Appl Environ Microbiol.

[33] Leandro MJ, Gonçalves P, Spencer-Martins I (2006). Two glucose/xylose transporter genes from the yeast Candida intermedia: first molecular characterization of a yeast xylose-H+ symporter. Biochem J.

[34] Ren L, Liu Y, Xia Y, Huang Y, Liu Y, Wang Y, Li P, Chang K, Xu D, Li F (2022). Improving glycerol utilization during high-temperature xylitol production with Kluyveromyces marxianus using a transient clustered regularly interspaced short palindromic repeats (CRISPR)/CRISPR-associated protein 9 system. Biores Technol.

[35] Liu N, Qiao K, Stephanopoulos G (2016). (13)C Metabolic flux analysis of acetate conversion to lipids by *yarrowia lipolytica*. Metab Eng.

[36] Cheng H, Wang S, Bilal M, Ge X, Zhang C, Fickers P, Cheng H (2018). Identification, characterization of two NADPH-dependent erythrose reductases in the yeast *Yarrowia lipolytica* and improvement of erythritol productivity using metabolic engineering. Microb Cell Fact.

[37] Singh A, Mishra P (1995). Microbial pentose utilization: current applications in biotechnology.

[38] Jafarihaghighi F, Ardjmand M, Salar Hassani M, Mirzajanzadeh M, Bahrami H (2020). Effect of fatty acid profiles and molecular structures of nine new source of biodiesel on combustion and emission. ACS Omega.

[39] Nielsen F, Zacchi G, Galbe M, Wallberg O (2017). Sequential targeting of xylose and glucose conversion in fed-batch simultaneous saccharification and co-fermentation of steam-pretreated wheat straw for improved xylose conversion to ethanol. BioEnergy Research.

[40] Madzak C, Tréton B, Blanchin-Roland S (2000). Strong hybrid promoters and integrative expression/secretion vectors for quasi-constitutive expression of heterologous proteins in the yeast *Yarrowia lipolytica*. J Mol Microbiol Biotechnol.

[41] Guo Z-p, Duquesne S, Bozonnet S, Nicaud J-M, Marty A, O’Donohue MJ (2017). Expressing accessory proteins in cellulolytic *Yarrowia lipolytica* to improve the conversion yield of recalcitrant cellulose. Biotechnol Biofuels.

[42] Xu P, Qiao K, Ahn WS, Stephanopoulos G (2016). Engineering *Yarrowia lipolytica* as a platform for synthesis of drop-in transportation fuels and oleochemicals. Proc Natl Acad Sci USA.

[43] Guo ZP, Duquesne S, Bozonnet S, Cioci G, Nicaud JM, Marty A, O'Donohue MJ (2017). Conferring cellulose-degrading ability to *Yarrowia lipolytica* to facilitate a consolidated bioprocessing approach. Biotechnol Biofuels.

[44] Blazeck J, Liu L, Knight R, Alper HS (2013). Heterologous production of pentane in the oleaginous yeast *Yarrowia lipolytica*. J Biotechnol.

[45] Xue Z, Sharpe PL, Hong S-P, Yadav NS, Xie D, Short DR, Damude HG, Rupert RA, Seip JE, Wang J (2013). Production of omega-3 eicosapentaenoic acid by metabolic engineering of *Yarrowia lipolytica*. Nat Biotechnol.

[46] Dulermo T, Tréton B, Beopoulos A, Kabran Gnankon AP, Haddouche R, Nicaud J-M (2013). Characterization of the two intracellular lipases of *Y. lipolytica* encoded by TGL3 and TGL4 genes: new insights into the role of intracellular lipases and lipid body organisation. Biochimica et Biophysica Acta BBA Mol Cell Biol Lipids.

[47] Borsenberger V, Onesime D, Lestrade D, Rigouin C, Neuveglise C, Daboussi F, Bordes F (2018). Multiple parameters drive the efficiency of CRISPR/Cas9-Induced gene modifications in yarrowia lipolytica. J Mol Biol.

[48] Reichenbecher M, Lottspeich F, Bronnenmeier K (1997). Purification and properties of a cellobiose phosphorylase (CepA) and a cellodextrin phosphorylase (CepB) from the cellulolytic thermophile *Clostridium* stercorarium. Eur J Biochem.

[49] Guo ZP, Olsson L (2016). Physiological responses to acid stress by *Saccharomyces* cerevisiae when applying high initial cell density. FEMS Yeast Res.

[50] Rolfe MD, Rice CJ, Lucchini S, Pin C, Thompson A, Cameron AD, Alston M, Stringer MF, Betts RP, Baranyi J (2012). Lag phase is a distinct growth phase that prepares bacteria for exponential growth and involves transient metal accumulation. J Bacteriol.

