# Supplementary material for: Installing xylose assimilation and cellodextrin phosphorolysis pathways in obese Yarrowia lipolytica facilitates cost-effective lipid production from lignocellulosic hydrolysates
Source: Biotechnol Biofuels Bioprod. 2023 Nov 29;16:186. doi: 10.1186/s13068-023-02434-9 (PMC10688077; doi:10.1186/s13068-023-02434-9)
Supplement: Supplementary file 1 — Additional file 1: Table S1 Plasmids used or created in the present study. Table S2 The sequences of the oligonucleotide primers used in this study. Table S3 sgRNA for gene deletion. Table S4 The composition of steam-pretreated wheat straw. [file 13068_2023_2434_MOESM1_ESM.docx]

Table S1 Plasmids used or created in the present study

| Plasmids | Description | Source of reference |
| --- | --- | --- |
| pYLXP | *URA3*, *LEU2*, *pTEF*-*tXPR* | [1] |
| pYL1 | *LEU2*, *pTEF*-*tXPR* | [1] |
| pYU1 | *URA3*, *pTEF*-*tXPR* | This investigation |
| pYL4 | *LEU2*, *p4UASTEF*-*tXPR* | This investigation |
| pYU4 | *URA3*, *p4UATEF*-*tXPR* | This investigation |
| pYL8 | *LEU2*, *p8UASTEF*-*tXPR* | This investigation |
| pYU8 | *URA3*, *p8UATEF*-*tXPR* | This investigation |
| pYL1-Ctcbp1 | *LEU2*, *pTEF-Ctcbp1-tXPR* | This investigation |
| pYL1-Ctcdp1 | *LEU2*, *pTEF-Ctcdp1-tXPR* | This investigation |
| pYL1-NcCDT1 | *LEU2*, *pTEF-NcCDT1-tXPR* | This investigation |
| pYL1-ScPGM2 | *LEU2*, *pTEF-ScPGM2-tXPR* | This investigation |
| pYL4-Ctcbp1 | *LEU2*, *p4UATEF-Ctcbp1-tXPR* | This investigation |
| pYL4-Ctcdp1 | *LEU2*, *p4UATEF-Ctcdp1-tXPR* | This investigation |
| pYL4-NcCDT1 | *LEU2*, *p4UATEF-NcCDT1-tXPR* | This investigation |
| pYL4-ScPGM2 | *LEU2*, *p4UATEF-ScPGM2-tXPR* | This investigation |
| pYL8-Ctcbp1 | *LEU2*, *p8UATEF-Ctcbp1-tXPR* | This investigation |
| pYL8-Ctcdp1 | *LEU2*, *p8UATEF-Ctcdp1-tXPR* | This investigation |
| pYL8-NcCDT1 | *LEU2*, *p8UATEF-NcCDT1-tXPR* | This investigation |
| pYL8-ScPGM2 | *LEU2*, *p8UATEF-ScPGM2-tXPR* | This investigation |
| pYL1-BDP | *LEU2*, *pTEF-Ctcbp1-tXPR, pTEF-Ctcdp1-tXPR, pTEF-NcCDT1-tXPR, pTEF-ScPGM2-tXPR* | This investigation |
| pYL4-BDP | *LEU2*, *p4UATEF-Ctcbp1-tXPR, p4UATEF-Ctcdp1-tXPR, p4UATEF-NcCDT1-tXPR, pTEF-ScPGM2-tXPR* | This investigation |
| pYL8-BDP | *LEU2*, *p8UATEF-Ctcbp1-tXPR, p8UATEF-Ctcdp1-tXPR, p8UATEF-NcCDT1-tXPR, pTEF-ScPGM2-tXPR* | This investigation |
| pYU1-SsXR | *URA3*, *pTEF-SsXR-tXPR* | This investigation |
| pYU1-SsXDH | *URA3*, *pTEF-SsXDH-tXPR* | This investigation |
| pYU1-CiGXF | *URA3*, *pTEF-CiGXF1-tXPR* | This investigation |
| pYU4-SsXR | *URA3*, *p4UATEF-SsXR-tXPR* | This investigation |
| pYU4-SsXDH | *URA3*, *p4UATEF-SsXDH-tXPR* | This investigation |
| pYU4-CiGXF | *URA3*, *p4UATEF-CiGXF1-tXPR* | This investigation |
| pYU1-XKS | *URA3*, *pTEF-XKS1-tXPR* | This investigation |
| pYU1-ZWF | *URA3*, *pTEF-ZWF1-tXPR* | This investigation |
| pYU1-GND | *URA3*, *pTEF-GND1-tXPR* | This investigation |
| pYU4-XKS | *URA3*, *p4UATEF-XKS-tXPR* | This investigation |
| pYU4-ZWF | *URA3*, *p4UATEF-ZWF1-tXPR* | This investigation |
| pYU4-GND | *URA3*, *p4UATEF-GND1-tXPR* | This investigation |
| pYU1-XRK | *URA3*, *pTEF-SsXR-tXPR, pTEF-SsXDH-tXPR* | This investigation |
| pYU4-XRK | *URA3*, *p4UATEF-SsXR-tXPR, p4UATEF-SsXDH-tXPR, p4UATEF-XKS-tXPR, p4UATEF-CiGXF1-tXPR, p4UATEF-ZWF1-tXPR, p4UATEF-GND1-tXPR* | This investigation |
| pYU1-GPD | *URA3*, *pTEF-GPD1-tXPR* | This investigation |
| pYL1-DGA | *LEU2*, *pTEF-DGA2-tXPR* | This investigation |

Table S2 The sequences of the oligonucleotide primers used in this study

| Primer | Sequence (5’-3’) | Restriction sites | Template | Product |
| --- | --- | --- | --- | --- |
| Vec1f | tatgctagcgagacaataacgga | *-* | pYL1/4/8 or pYU1/4/8 | Plasmid backbone |
| Vec1r | acgaagttatcctaggctttcatc | *-* |  |  |
| ASS1f | cctaggataacttcgggttggcggcgcatttgtgt | *-* | pYL1-Ctcbp1, pYU1-SsXR, pYL4-Ctcbp1, pYU4-SsXR | pTEF-Ctcbp-tXPR, pTEF-SsXR-tXPR, |
| ASS1r | caccttcgatttcgaggacacgggcatctcacttgcat | *-* |  | p4UASTef-Ctcbp-tXPR, p4UASTef-SsXR-tXPR |
| ASS2f | tcgaaatcgaaggtgggttggcggcgcatttgtgt | *-* | pYL1-Ctcdp1, pYU1-SsXDH, pYL4-Ctcdp1, pYU4-SsXDH | pTEF-Ctcdp-tXPR, pTEF-SsXDH-tXPR,  p4UASTef-Ctcdp-tXPR, p4UASTef-SsXDH-tXPR |
| ASS2r | gtctaccctcaccttggacacgggcatctcacttgcat | *-* |  |  |
| ASS3f | aaggtgagggtagacggttggcggcgcatttgtgt | *-* | pYL1-NcCDT1, pYU1-XK, pYL4-NcCDT1, pYU4-XK | pTEF-NcCDT1-tXPR, pTEF-XK-tXPR,  p4UASTef-NcCDT1-tXPR, p4UASTef-XK-tXPR |
| ASS3r | ctgtttgagtacctgggacacgggcatctcacttgcat | *-* |  |  |
| ASS4f | aggtactcaaacagcggttggcggcgcatttgtgt | *-* | pYL1-PGM, pYU1-GXF | pTEF-PGM2-tXPR, pTEF-GXF1-tXPR |
| ASS4r | tgtctcgctagcataggacacgggcatctcacttgcat | - |  |  |
| XRf | gc*tctaga*cacaatgccttctattaagttgaactctggt | *Xba*I | Genomic DNA of *S. stipites* | *SsXR* |
| XRr | gg*ggtacc*ttagacgaagataggaatcttgtccc | *Kpn*I |  |  |
| XDHf | gc*tctaga*cacaatgactgctaacccttccttggtgttg | *Xba*I |  | *SsXDH* |
| XDHr | gg*actagt*ttactcagggccgtcaatgagacac | *Spe*I |  |  |
| PGMf | gc*tctaga*cacaatgtcatttcaaattgaaacggttccca | *Xba*I | Genomic DNA of *S. cerevisiae* | *ScPGM2* |
| PGMr | gg*actagt*ttaagtacgaaccgttggttcttcagttcc | *Spe*I |  |  |
| XKf | gc*tctaga*cacaatgtatctcggactggatctttcgactc | *Xba*I | Genomic DNA of *Y. lipolytica* | *YlXK* |
| XKr | gg*ggtacc*ttatttctccaggcaggcgttttc | *Kpn*I |  |  |
| ZWFf | gc*tctaga*cacaatgactggcaccttacccaagtt | *Xba*I |  | *YlZWF1* |
| ZWFr | gg*ggtacc*tcacgaggagcccttggtg | *Kpn*I |  |  |
| GNDf | gc*tctaga*cacaatgactgacacttcaaacatcaag | *Xba*I |  | *YlGND1* |
| GNDr | gg*ggtacc*ttaagcatcgtaagtggaagaag | *Kpn*I |  |  |
| GPDf | gg*ggtacc*atgagcgctctacttcgatcgt | *Kpn*I |  | *YlGPD1* |
| GPDr | gg*actagt*tagttggcgtggtaaagaatctcgggg | *Spe*I |  |  |
| DGAf | gc*tctaga*atggaagtccgacgacgaaaaatc | *Xba*I |  |  |
| DGAf | gc*tctaga*atggaagtccgacgacgaaaaatc | *Kpn*I |  | *YlDGA2* |
| VPEX10f | atgtggggaagttcacatgcattc |  | gDNA of *Y. lipolytica* po1f-1 (ΔMEF1, ΔPEX10, ΔTGL4) | Disrupted *PEX10* (637 bp) |
| VPEX10r | ttatctgataggcaacaagttctgctct |  |  |  |
| VMFE1f | atgtctggagaactaagatacgacgg |  |  | Disrupted *MEF1* (2321 bp) |
| VMFE1r | ttagagcttagcatccttggggaag |  |  |  |
| VTGL4f | atgttcacctccagagtttccg |  |  | Disrupted *TGL4* (1417 bp) |
| VTGL4r | ttagcacgagtcagaacagttctc |  |  |  |

Restriction sites are italic/underlined.

Homologous sequence for infusion or recombination is double underlined.

Table S3 sgRNA for gene deletion

| Target gene | The sequence of sgRNA (5’-3’) |
| --- | --- |
| *PEX10* | *AGACCGAACAGCTCTACCGG* (386 fwd) |
|  | CCTTCTGTATCCTCAATGAA (886 rev) |
| *MFE1* | GTCTGCTATTCTGAAGCGAT (901 fwd) |
|  | CTTAAGGAAGTGGGGCCACG (1363 rev) |
| *TGL1* | ACTTTATCCGGCCGACGGCA (58 fwd) |
|  | CCGACCAAATGAGTACGTTG (1095 rev) |

Table S4 The composition of steam-pretreated wheat straw

| Component | Content |
| --- | --- |
| WIS content (wt%) | 12±1.2 |
| Composition of WIS (wt%) |  |
| Glucan | 48.9±1.4 |
| Xylan | 1.4±0.5 |
| Galactan | - |
| Arabinan | 0.08±0.03 |
| Mannan | 0.06±0.02 |
| Composition of hydrolysate |  |
| Glucose | 8.6±0.9 |
| Xylose | 33.5±1.7 |
| Galactose | 1.2±0.4 |
| Arabinose | 2.2±0.8 |
| Mannose | 1.0±0.3 |
| Formic acid | 0.9±0.1 |
| Levulinic acid | 0.6±0.2 |
| Acetic acid | 4.8±0.6 |
| HMF | 0.4±0.1 |
| Furfural | 2.9±0.2 |

± the standard deviation.

**Lipid accumulation on glucose**


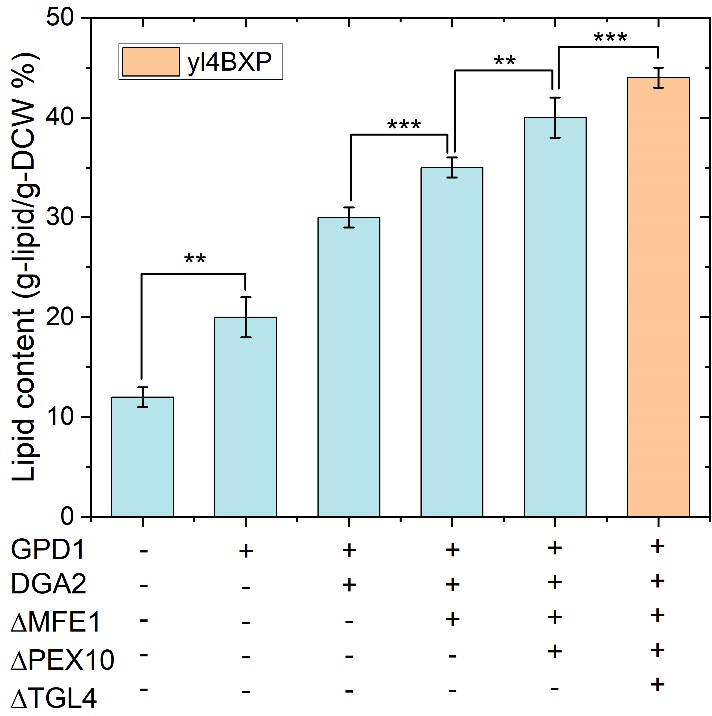
 The preculture was carried out in YNB media and used to inoculate 1.2L YNB media containing 75 g/L glucose in a 3.0-liter stirred-tank bioreactor (Sartorius, Germany) to reach an initial OD600nm of 1.0. A feeding of 75 g/L of glucose was performed when the concentration of total reducing sugars dropped below 10 g/L. NH_4_Cl was added into the media at the concentration to yield a C/N ratio of 60:1. Throughout the fermentation process, the pH was maintained at 5.5 with the automatic addition of 2.0 M NaOH and the temperature was kept at 28°C. An aeration of 0.5 vvm was set up and the stirring speed was automatically controlled to keep the dissolved oxygen at 20% of air saturation. Samples were taken regularly to analyze the concentrations of biomass, metabolites and carbon source in culture media.

Fig. 2 Comparsion of cellular lipid content of recombinant strains of *Y. lipolytica* constructed in this work. The strategy used to increase lipid accumulation including overexpress *GPD1* and *DGA2*, and interruption of *MFE1*, *PEX10* and *TGL4*. (** P value<0.05, ***P value<0.01, two-tailed Student’s t-tests).

Reference

1. Xu P, Qiao K, Ahn WS, Stephanopoulos G: **Engineering Yarrowia lipolytica as a platform for synthesis of drop-in transportation fuels and oleochemicals**. *Proc Natl Acad Sci U S A* 2016, **113**(39):10848-10853.
